# Remote ECG Monitoring by ECG247 Smart Heart Sensor

**DOI:** 10.1155/2022/6812889

**Published:** 2022-02-11

**Authors:** Jarle Jortveit, Rune Fensli

**Affiliations:** ^1^Sorlandet Hospital, Department of Cardiology, Arendal, Norway; ^2^Faculty of Engineering and Science, University of Agder, Grimstad, Norway

## Abstract

**Background:**

Heart rhythm disorders are common and may be associated with serious complications. The quality of the ECG signal is crucial to detect and classify arrhythmias. Most available devices for assessment arrhythmias do not allow for remote monitoring. The Norwegian ECG247 Smart Heart Sensor is a new remote patch monitor developed to simplify the assessment of arrhythmias. This study was aimed at evaluating the quality of the ECG signal from the ECG247 Smart Heart Sensor compared to standard 12-lead ECG.

**Methods:**

ECG recordings with ECG247 Smart Heart Sensor and a standard 12-lead ECG recorder were performed in 97 volunteers at Sorlandet Hospital, Arendal, Norway, in 2019. All ECGs were analysed by two independent cardiologists.

**Results:**

A total of 97 participants (53% men, age 48 (±14) years) were included in the study. The ability for both systems to use recorded ECG data for arrhythmia detection was good (100%). The quality of the P-wave (mean score 1.1 vs. 1.5) and the QRS complex (mean score 1.0 vs. 1.0) from the ECG247 Smart Heart Sensor and that from the 12-lead ECG were comparable (scale: 1: extremely good, 9: not accepted). Noise artefacts were a minor issue in all recordings.

**Conclusions:**

The ECG quality from the ECG247 Smart Heart Sensor was comparable to the ECG quality from the standard 12-lead ECG. The ECG247 Smart Heart Sensor may enable easy and remote diagnostics of heart rhythm disorders. This trial is registered with NCT04700865.

## 1. Introduction

Heart rhythm disorders may cause severe complications, e.g., death, stroke, and heart failure [1]. A correct diagnosis is important to prevent mortality and morbidity. Long-term ECG recording increases the ability to detect intermittent arrhythmias [2]. Several systems for long-term ECG monitoring are in routine clinical use in the health care system. A conventional Holter monitor system requires a recording device coupled to electrodes on the chest [3]. Such systems may be cumbersome to use, have limited test period, and are sensitive to disturbances. “Thumb ECG” systems have very short ECG recording time and consequently lower sensitivity to intermittent arrhythmias. Implantable loop recorders allow for continuous rhythm monitoring over years but require surgical procedures. Limited number of devices for long-term ECG recording at many hospitals due to high purchasing and operation costs may lead to delays in assessment and treatment. ECG documentation of arrhythmias is necessary, and watches with heart rate monitoring based on arterial pressure waves cannot be used for clinical diagnostics [4]. Wearable ECG patch devices may facilitate diagnosis of heart rhythm disorders [5]. However, high usability for both patients and health care professionals, low cost, continuous heart rhythm monitoring, and remote access to ECG recordings are preferable.

The ECG247 Smart Heart Sensor is a new Norwegian telemedicine technology for remote diagnostics of heart rhythm disorders. The diagnostic accuracy of the ECG247 Smart Heart Sensor automatic arrhythmia detection algorithm is recently presented [6]. However, the quality of the ECG signal is crucial to detect arrhythmias and to minimize the number of false-positive test results. Furthermore, a single lead ECG may be more difficult to analyse for arrhythmias than a multilead ECG.

The objectives of this study were to assess the quality of the ECG247 Smart Heart Sensor ECG recordings and to evaluate the possibility of using the actual 1-lead ECG recordings for determining the heart rhythm.

## 2. Methods

### 2.1. Study Design

This prospective feasibility study was conducted at Sorlandet Hospital Arendal, Norway, between April 2019 and August 2019.

### 2.2. Study Population

A total of 97 volunteers ≥ 18 years without prior heart rhythm disorders were recruited to participate in the study.

### 2.3. Diagnostic Devices

**Figure 1:**
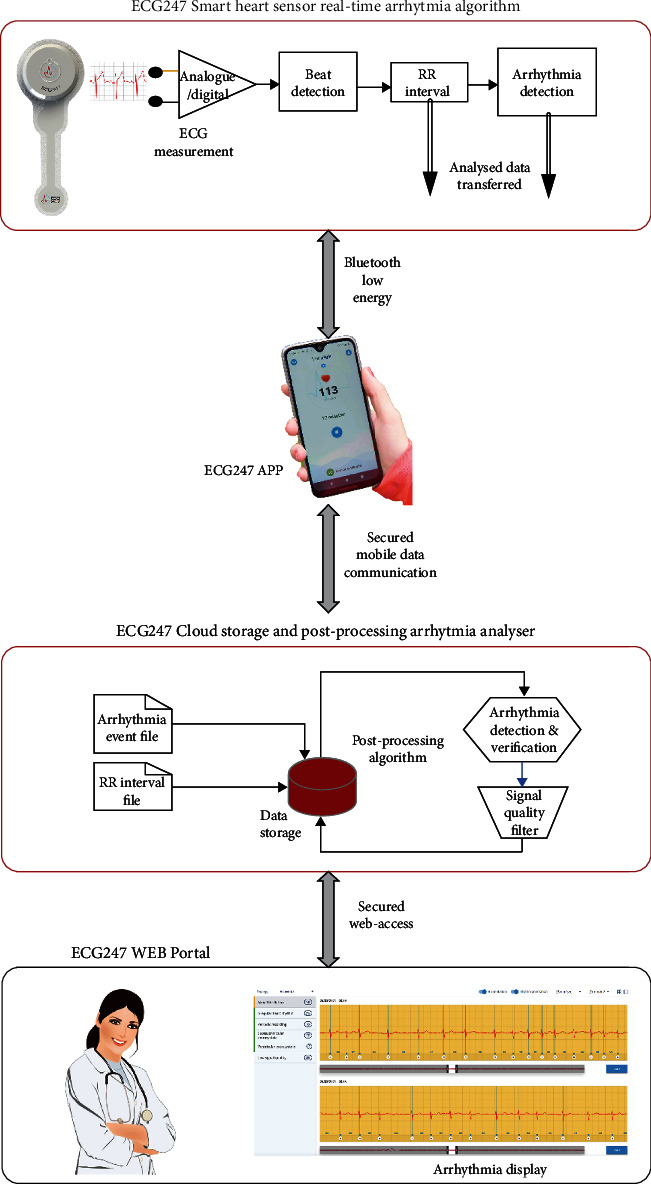
The ECG247 Smart Heart Sensor system: sensor with real-time arrhythmia detection, smartphone application, back-end cloud service with postprocessing arrhythmia analyser, and web portal.

The index tests were performed with the ECG247 Smart Heart Sensor (AppSens, Lillesand, Norway). The ECG247 Smart Heart Sensor is a water-resistant easy-to-use wireless single-lead continuous ECG monitoring system consisting of an electrode patch, a reusable sensor, a smartphone application, a back-end cloud service, and a web portal (Figure 1). The system follows the requirements given by the General Data Protection Regulation (GDPR) and is certified as a medical diagnostic device according to the EU Medical Device Directives (93/42/EEC).

The ECG247 Smart Heart Sensor system is built on the requirements given by the international standard IEC 60601-2-47 which defines the requirements for the basic safety and essential performance of ambulatory electrocardiographic systems. ECG recordings are made with a sampling frequency of 256 Hz and a signal resolution of 16 bits, with a bandwidth of 0.5 Hz to 40 Hz. The sensor has incorporated a flash memory for temporary storage of ECG data in case of temporary loss of Bluetooth communication with the user's smartphone.

A patented dedicated shielding system is incorporated into the ECG247 electrode patch to protect the ECG pick-up electrodes from electrostatic discharges from clothes [7]. Based on principles from a Faraday cage, a thin metallic foil is placed directly above the two ECG pick-up electrodes and connected to the patient's skin by a third “grounding” electrode. By this unique design of the sensor, a noise-free ECG signal can be obtained even if the patient is wearing a shirt rubbing against the sensor surface during daily activities.

The sensor transmits recorded ECG-recorded data by Bluetooth Low Energy (BLE) to a smartphone with the ECG247 mobile application installed. The app transmits the recorded ECG data to a secured cloud-based storage (Microsoft Azure), where the doctor can log in having real-time access to the patient's data. To guarantee for cyber security and for the quality of service (QoS) in the wireless communication, Bluetooth is protected by an encryption protocol and a frequently changing BLE GAP address. ECG data files are coded according to the international standard ISO/TS 22077-3 and are transferred from the smartphone to the back-end services as data packages containing 1 minute of ECG recordings, giving 15,360 data samples. In cases of detected package loss received by the back-end services, this is automatically retransmitted, and the revised file is controlled for completeness. Based on the incorporated features, the system has no strict requirements to QoS for bandwidth, error rate, delay, or latency in the wireless communication.

The ECG247 Smart Sensor system continuously analyses the heart rhythm for arrhythmias by use of Artificial Intelligence (AI) with high diagnostic accuracy as previously described [6].

A standard 12-lead ECG recorded with a Schiller Cardiovit AT-102 G2 was used as the reference standard in the study.

### 2.4. Study Procedure

**Figure 2:**
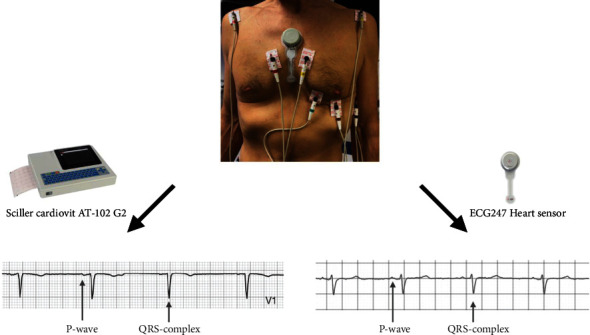
Parallel ECG recordings with ECG247 Smart Heart Sensor and Schiller Cardiovit AT-102 G2 ECG recorder.

Parallel tests with the ECG247 Smart Heart Sensor and the standard 12-lead ECG were performed at rest in all study patients. Both systems were mounted by a study nurse, and ECG recordings were saved simultaneously (Figure 2). All ECGs were reviewed as a visual test of recorded printouts performed by two independent and experienced cardiologists. For each record, the cardiologists filled in a questionnaire defining the quality of the P-wave, the quality of the QRS complex, noise artefacts, and the possibility to determine the heart rhythm.

### 2.5. Outcomes

The primary outcome was the ability to determine the heart rhythm at ECGs recorded with the ECG247 Smart Heart Sensor system. The secondary outcomes were the quality of the P-wave and the QRS complex in the ECG247 Smart Sensor ECGs compared with the standard 12-lead ECGs, as well as the presence of noise artefacts.

### 2.6. Statistics

Continuous variables are presented as means ± standard deviation (SD) or medians. Independent sample *t*-tests were used to analyse differences between groups. Categorical variables are presented as numbers and percentages. Sample sizes between 24 and 50 have been recommended for feasibility studies [8]. STATA version 16 (StataCorp LLC, College Station, TX, USA) was used for data analysis.

### 2.7. Patient and Public Involvement

The study protocol was designed in close cooperation with user representatives.

### 2.8. Ethics

The study was approved by the Regional Committee for Medical and Health Research Ethics (REK 2018/1854). The consent form was signed by all participants.

## 3. Results

A total of 97 volunteers were included in the study at Sorlandet Hospital, Arendal, Norway, between April 2019 and August 2019. Clinical characteristics are reported in Table 1.

The result of the cardiologist assessments of the ECG quality is reported in Table 2. The heart rhythm was possible to determine in all cases. All participants had normal sinus rhythm. We found comparable quality of the P-waves and the QRS complexes between the ECG247 Smart Heart Sensor and the 12-lead ECG recordings. Noise artefacts were a minor issue in all recordings.

## 4. Discussion

This study of the ECG247 Smart Heart Sensor versus the standard 12-lead ECG demonstrated comparable ECG quality and ability to determine the heart rhythm.

The key to improving the diagnostic yield in arrhythmias is the quality of the ECG signal. Clarity of the P-waves and the QRS complexes, their morphology, their relationship, and timing are crucial for defining arrhythmias. Similarly, electrical “noise” may be misidentified as cardiac beats and may interfere with the diagnostic of arrhythmias. Even if the ECG-recordings from the ECG247 Smart heart Sensor is only one-lead ECG and differs in shape of the recorded ECG curve compared to the standard 12-lead ECG, the analysed recordings showed good signal quality and equally high score for rhythm detection compared to the referenced standard.

This may be caused by the unique design in the wireless ECG recording sensor.

Some patients found wired ambulatory ECG monitors uncomfortable to wear [9, 10]. High usability of the ECG247 Smart Heart Sensor allows for long heart rhythm monitoring, which increases the diagnostic yield [6, 11].

The European Society of Cardiology guidelines recommend screening for atrial fibrillation in people at increased risk of stroke [4]. However, the number of devices for long-term continuous ECG recording is limited [12]. The ECG247 Smart Heart Sensor enables remote screening for atrial fibrillation with low cost. The sensor can be self-applied, and all ECG recordings are remotely available in real time for health care professionals. The coronavirus disease 2019 (COVID-19) pandemic also underlines the need for medical diagnostics away from the patient.

This study has several limitations. It was a single-center study with relatively few participants. Moreover, none of the participants had any arrhythmia. All ECG recordings were stored at rest. Physical activity can interfere with the quality of ECG recordings.

In conclusion, the ECG247 Smart Heart Sensor had comparable ECG quality compared to standard 12-lead ECG and allow for easy-to-use remote diagnostic of heart rhythm disorders by telemedicine technology.

## Figures and Tables

**Table 1:** Clinical characteristics.

	*n* = 97
Women (*n*)	46 (47%)
Mean age ± SD (year)	48 (14)
Mean height ± SD (cm)	171 (27)
Mean weight ± SD (kg)	76 (14)
Median BMI (min, max)	24 (16, 37)

**Table 2:** ECG quality score^∗^ and the possibility to determine heart rhythm.

	12-lead ECG	ECG247 Smart Heart Sensor ECG	*p*
Rhythm detection	97 (100%)	97 (100%)	1.0

P-wave (mean score ± SD)	1.5 (0.7)	1.1 (0.5)	<0.001

QRS complex (mean score ± SD)	1.0 (0.0)	1.0 (0.0)	1.0

Noise artefacts (mean score ± SD)	1.1 (0.2)	1.3 (0.4)	<0.001

^∗^1: extremely good; 9: not accepted.

## Data Availability

The data that support the findings of this study are available on request from the corresponding author.

## Additional Points

*Study Protocol*. Available on request from the corresponding author.
